# Experience in the management of real-world patients with chronic prurigo nodularis in a dermatology setting: results from the ECOSPIN Spanish survey-based study

**DOI:** 10.3389/fmed.2026.1769149

**Published:** 2026-04-14

**Authors:** Ignasi Figueras-Nart, Rafael Ortiz Castillo, Esther Serra Baldrich

**Affiliations:** 1 Department of Dermatology, Hospital Universitari de Bellvitge, University of Barcelona, Barcelona, Spain; 2 Sanofi S.A., Madrid, Spain; 3 Department of Dermatology, Sant Pau Hospital, Barcelona, Spain

**Keywords:** chronic prurigo nodularis, real-world, skin diseases, survey, treatment, burden of CPN (Chronic prurigo nodularis)

## Abstract

**Background:**

Chronic prurigo nodularis (CPN) is a chronic, inflammatory, highly pruritic skin disease with a significant burden, but the epidemiological data available are still very limited. This study aimed to describe the sociodemographic and clinical profiles of real-world patients with CPN seen in dermatology consultations in Spain.

**Methods:**

ECOSPIN was a Spanish, observational, ecological, survey-based study with aggregated data obtained from the experience and routine clinical practice of 39 dermatologists.

**Results:**

According to the dermatologists, a mean of 29.1% of patients with CPN were aged 51–60 years, and the majority were women (61%). A mean of 97.9% experienced itching, with frequent comorbidities: atopic dermatitis (41.1%), hypertension (32.1%), anxiety (57.4%), and depression (43.6%). Dermatologists considered the etiology of CPN to be dermatological in a mean of 56.5% of cases. The primary treatments were topical corticosteroids and calcineurin inhibitors (91.2%). Among systemic therapies, dupilumab was the most used (51.8%) and was rated as the most effective. The most relevant treatment goal was rapid itch elimination or reduction, while managing comorbidities was important for minimizing healthcare resource utilization, based on the dermatologists’ experience.

**Conclusion:**

The study highlighted unmet needs and the high burden of CPN, emphasizing the need for multidisciplinary and individualized treatment approaches.

## 1 Introduction

Chronic prurigo nodularis (CPN), also named prurigo nodularis, is a relatively uncommon chronic, inflammatory, highly pruritic skin disease characterized by the presence of multiple hyperkeratotic and fibrotic papules and nodules that are usually symmetrically distributed on the limbs and trunk (1, 2). CPN is a subtype of chronic prurigo, a distinct disease defined by the presence of chronic pruritus and multiple pruriginous lesions that are either localized or widespread. CPN is increasingly recognized as a systemic inflammatory disease, characterized by immune dysregulation and neuronal sensitization (3). The origin of CPN has been associated with dermatologic conditions, such as atopic dermatitis (AD) (4); systemic diseases, such as chronic renal failure, liver disease, thyroid disease, diabetes, or infections (5); and neurological and psychiatric disorders (6). However, although some of these conditions may be causative, the precise relationship remains unknown. CPN seems to be more common among older adults (with a mean age of 50–55 years), women, and African Americans (5, 7, 8). However, epidemiological data on CPN as a disease entity are very limited.

CPN is associated with a significant disease burden, including sleep impairment, anxiety, and depression, and it severely impacts patients’ quality of life (9). CPN also has a notable economic impact, requiring more medical visits than many other skin diseases and a higher cost of hospital care (10). The management of CPN is highly complex because of the multiple underlying causes and clinical presentations. Currently, several therapies are prescribed for CPN management, including topical therapies, phototherapy, immunosuppressive therapy, systemic neuromodulators, and antidepressants, but the efficacy of these therapies is generally low and/or associated with adverse effects (11).

Progress in the understanding of CPN pathophysiology and the identification of specific therapeutic targets have led to the development of promising new drugs that may improve the management of this difficult-to-treat disease. Recent research has revealed that T-helper 2 (Th2) cytokines, such as interleukin-4 (IL-4), interleukin-13 (IL-13), and interleukin-31 (IL-31), may play a role in CPN pathogenesis (12). Dupilumab, a monoclonal antibody that blocks IL-4/13 signaling, has been well tolerated and has demonstrated clinically and statistically significant improvements in itch and skin lesions in patients with CPN in randomized phase 3 clinical trials (13). Similarly, nemolizumab, a monoclonal antibody targeting the interleukin-31 (IL-31) receptor, has exhibited reductions in disease burden in patients with CPN in randomized phase 3 clinical trials (14). Initially approved in 2017, dupilumab indications were expanded in Europe in 2022, making it the first targeted therapy approved for the treatment of moderate-to-severe CPN in adult patients (15). In 2025, nemolizumab for CPN was also granted approval in Europe (16).

However, reports based on dermatologists’ experience regarding the CPN patient profile, disease burden, and diagnosis and management of CPN require further exploration. As access to new therapies becomes available, there is an opportunity to reassess and refine treatment goals. A physician survey of 30 clinicians from 14 countries, including European countries, addressed questions about the definition and terminology of CPN, its prevalence, patient demographics and disease burden, and approaches to its diagnosis and treatment (17). Additionally, to describe CPN management, a web-based survey was conducted among Japanese dermatologists (18). Nonetheless, as a result of the differences in epidemiology and local clinical practice among countries, real-world data from various healthcare systems may be heterogeneous. In this context, the current study aimed to describe the sociodemographic and clinical profile of real-world patients with CPN in dermatology consultations in Spain.

## 2 Materials and methods

### 2.1 Study population and design

ECOSPIN was a Spanish, observational, ecological, survey-based study using aggregated data. Data were collected based on dermatologists’ insights in a real clinical setting, without reviewing patients’ medical records. Participants were selected based on their expertise, clinical experience, and the volume of patients attending their consultations. Dermatologists with at least 5 years of experience who had seen a mean of three patients with CPN per month over the past year were invited to participate in the study. To ensure a representative sample, 40 participating dermatologists were selected from hospitals across various regions of Spain.

### 2.2 Survey and data collection

A total of 39 dermatologists completed an online survey between November and December of 2024. The participants are listed in the Supplementary Table. Each dermatologist reported their experience with the last five patients diagnosed with CPN attending their consultation. In cases where participants indicated that they did not have data for a question, the number of participants who responded with data is specified for that question. The survey (Supplementary Data) consisted of 42 questions divided into two sections. The first 9 questions addressed the characteristics of the participants, while the remaining 33 questions were oriented to describe the sociodemographic and clinical profile of patients with CPN (14 questions), the impact of disease burden on patients (4 questions), the management of CPN (10 questions), and healthcare resources according to the participants’ knowledge and experience in routine clinical practice (5 questions). Completion of the survey required responses to all questions. All participants received remuneration from the sponsor after completing the survey. Patient classification using the Investigator’s Global Assessment (IGA) was based on the proportion of pruritic lesions exhibiting excoriations or scabbing. The IGA categories were defined as follows: clear (score 0), no pruritic lesions exhibiting excoriations or scabs; almost clear (score 1), excoriations or scabs present in up to approximately 10% of pruritic lesions; mild (score 2), excoriations or scabs present in approximately 11–25% of pruritic lesions; moderate (score 3), excoriations or scabs present in approximately 26–75% of pruritic lesions; and severe (score 4), excoriations or scabs present in approximately 76–100% of pruritic lesions. The severity of pruritus was assessed using the Worst Itch Numeric Rating Scale (WI-NRS), based on the worst level of itch experienced in the previous 24 h, where 0 indicates no itch and 10 indicates the worst itch imaginable.

### 2.3 Statistical analysis

A descriptive analysis of the responses to the study was conducted. The frequency distributions of the qualitative variables were calculated and presented as absolute percentages. For quantitative variables, the measures of central tendency are presented as mean and standard deviation (SD), and the measures of dispersion are reported as median, interquartile range (IQR) [Q1–Q3], minimum, and maximum. The Statistical Package for the Social Sciences (SPSS) version 29.0 (SPSS Inc., Chicago, IL, USA) was used for data analysis.

## 3 Results

### 3.1 Dermatologists’ profile

A total of 39 dermatologists completed the survey, with a mean age of 47.5 (SD 9.2) years. Approximately half (51.3%) of them had 20 or more years of experience in dermatology. Participants saw a mean of 112.7 (SD 45.6) patients per week, of whom 3.4% (SD 3.4) were diagnosed with CPN, and a mean of 19.1 (SD 11.9) new cases in the last 12 months (Table 1). Overall, 71.8% of the participants practiced in public hospitals across Spain, and 28.2% worked in both public and private hospitals.

**Table 1:** Profile of the dermatologists participating in the ECOSPIN study.

Number of dermatologists	*N* = 39
Age, mean (SD)	47.5 (9.2)
Sex, *n* (%)	
Female	20 (51.3)
Male	19 (48.7)
Years of experience, *n* (%)	
5–10 years	8 (20.5)
11–19 years	11 (28.2)
20 or more years	20 (51.3)
Mean number (SD) of patients seen each week	112.7 (45.6)
Mean % (SD) of patients diagnosed with CPN of the total number of patients seen	3.4 (3.4)
Mean number (SD) of new CPN cases seen in the last 12 months	19.1 (11.9)

SD, standard deviation; CPN, chronic prurigo nodularis.

### 3.2 Patients’ characteristics

Patients’ characteristics reported by the participant dermatologists are summarized in Table 2. Considering the last 5 patients with CPN seen by the participants, a mean of 29.1% (SD 18.8) of the patients were in the 51–60 age range, and 61% (SD 18.9) were women. At the time of diagnosis, a mean of 84% (SD 23.3) of the patients had excoriations, 84% (SD 22.8) had scars, 82.3% (SD 21.9) had nodules, and 73.6% (SD 28.8) had papules. Dermatologists reported that a mean of 97.9% (SD 5.7) of the patients experienced itching. On average, 76.5% (SD 25.9) of the lesions were localized in the anterior lower legs, and 74.6% (SD 27.9) were localized in the upper back (Table 2). Anxiety and depression were present in a mean of 57.4% (SD 24.7) and 43.6% (SD 21.2) of the patients seen by the participants, respectively. According to the physicians’ experience, CPN was of dermatological etiology for a mean of 56.5% (SD 22.2) of the patients, followed by psychological or psychosomatic etiology in 30.3% (SD 21.1).

**Table 2:** Characteristics of the patients with CPN.

Number of dermatologists answering these questions	*N* = 39^1^
Age, mean % (SD)	
>81 years	2.7 (5.2)
71–80 years	8.8 (11.2)
61–70 years	19.6 (18.1)
51–60 years	29.1 (18.8)
41–50 years	21.1 (17.6)
31–40 years	10.6 (9.8)
21–30 years	4.7 (8.4)
Less than 20 years	3.5 (6.2)
Sex, mean % (SD)	
Female	61 (18.9)
Male	39 (18.9)
Family history of CPN, mean % (SD)	1.9 (4.8)^2^
Mean duration of disease since diagnosis in years, mean (SD)	4.2 (3.8)^3^
Clinical signs at the time of diagnosis, mean % (SD)	
Excoriations	84 (23.3)
Scars	84 (22.8)
Nodules	82.3 (21.9)
Papules	73.6 (28.8)
Depigmentation	64 (33.1)
Lichenification	59.5 (30.6)
Ulcerative lesions	35.1 (32.1)
Plaque lesions	33.3 (24.4)
Others	12.3 (28.5)
Symptoms at the time of diagnosis, mean % (SD)	
Itching	97.9 (5.7)
Sleep loss	78.7 (26.1)
Burning	60.9 (33.6)
Anxiety	57.4 (24.7)
Pain	51.7 (30.1)
Depression	43.6 (21.2)
Lesion location, mean % (SD)	
Front of the legs	76.5 (25.9)
Upper back	74.6 (27.9)
Front of the forearms	67.5 (31.3)
Back of the legs	59.5 (27.9)
Back of the forearms	59.3 (32.3)
Abdomen	50.3 (26.5)
Hands	17.8 (19.3)
Scalp	7.7 (10.2)
Face	7.6 (10.7)
Number of nodules, mean (SD)	53.9 (32.7)^4^
CPN etiology, mean % (SD)	
Dermatological	56.5 (22.2)
Psychological/psychosomatic	30.3 (21.1)
Systemic	20.4 (16.6)
Unknown	14 (15.4)
Medication	8.1 (20.6)
Neurological	3.9 (8.2)
Tumoral	3.3 (6.5)
Atopic comorbidities, mean % (SD)	
Atopic dermatitis	41.1 (21.7)
Allergic rhinitis	16.3 (14.9)
Asthma	13.4 (11.1)
Allergic conjunctivitis	9 (11.4)
Chronic obstructive pulmonary disease	6 (9.8)
Chronic rhinosinusitis with nasal polyps	3.4 (8.1)
Chronic spontaneous urticaria	3.4 (6.5)
Bullous pemphigoid	2 (8.1)
Eosinophilic esophagitis	0.2 (0.8)
Non-atopic comorbidities, mean % (SD)	
Hypertension	32.1 (17.8)
Dyslipidemia	28.1 (16.3)
Obesity	28 (17.9)
Metabolic syndrome	26 (20.4)
Cardiovascular disease	21.8 (14)
Nephropathy	10.8 (9.7)
Gastrointestinal disorders	4.6 (7.9)
IGA score, mean % (SD), activity^5^/stage^6^	
0—clear	2.9 (9.5)/3.2 (8.5)
1—almost clear	9.2 (11.6)/9.8 (12.1)
2—mild	13.7 (16.2)/17.7 (17.1)
3—moderate	35.4 (15.9)/42.7 (17.8)
4—severe	38.8 (27.9)/26.6 (22.3)
WI-NRS, mean % (SD)	
Range 0–3	12.6 (16.5)
Range 4–6	30.9 (19.5)
Range 7–10	56.5 (26.2)

SD, standard deviation; CPN, chronic prurigo nodularis; IGA, Investigator’s Global Assessment; WI-NRS, Worst Itch Numeric Rating Scale. ^1^Number of dermatologists answering these questions, unless stated otherwise; ^2^*N* = 24; ^3^*N* = 36; ^4^*N* = 30; ^5^*N* = 38; ^6^*N* = 36. IGA classification: clear, no pruritic lesions have excoriations or scabs; almost clear, up to approximately 10% of all pruritic lesions have excoriations or scabs; mild, approximately 11–25% of all pruritic lesions have excoriations or scabs; moderate, approximately 26–75% of all pruritic lesions have excoriations or scabs; severe, approximately 76–100% of all pruritic lesions have excoriations or scabs. WI-NRS classification: 0 indicates no itch, and 10 indicates the worst itch imaginable.

The most frequent atopic comorbidities reported were AD, affecting a mean of 41.1% (SD 21.7) of the patients, and allergic rhinitis, reported in 16.3% (SD 14.9). Arterial hypertension and dyslipidemia were the most common non-atopic comorbidities, which were present in a mean of 32.1% (SD 17.8) and 28.1% (SD 16.3) of the patients, respectively (Table 2). Based on the WI-NRS at the time of diagnosis, a mean of 56.5% (SD 26.2) of the patients were classified into the 7–10 range of severity. The IGA for CPN-activity score was classified as severe in a mean of 38.8% (SD 27.9) of the patients and 26.6% (SD 22.3) for CPN-stage (*N* = 38 and *N* = 36, respectively), according to the participants (Table 2). Dermatologists considered that concomitant psychiatric disorders were the most relevant factor in the prognosis and progression of CPN, with a mean score of 8.4 (SD 2.1) on a 1-to-10 scale, in which 10 represents the most important factor, followed by the severity of the disease (7.8 [SD 2.4]), comorbidities (6.8 [SD 2.2]), type of lesions and symptoms (6.7 [SD 2.3] and 6.2 [SD 2.4], respectively), age (5.1 [SD 2.5]), and genetic factors (3.9 [SD 2.2]).

### 3.3 Impact of morbidity burden on patients with CPN and direct healthcare resources

Concerning the impact of morbidity burden on patients with CPN, a mean of 44.7% (SD 23.2) of the patients required psychological support (*N* = 36), 87.2% (SD 17.5) experienced poor quality of life due to issues related to CPN, and 77.1% (SD 21.3) experienced sleep disturbances (*N* = 38). In a mean of 48.2% (SD 40.3) of cases, quality of life was assessed using questionnaires or scales in clinical practice (*N* = 37). In the past year, a mean of 26.5% (SD 19) of the patients required additional non-planned visits due to the progression of CPN (*N* = 37), 1.6% (SD 5.4) were hospitalized due to CPN, and 42.5% (SD 24.3) needed visits to other healthcare providers (*N* = 34). Moreover, a mean of 38.4% (SD 20) of the patients required psychological support (*N* = 37), as reported by the participants.

### 3.4 CPN management

**Figure 1:**
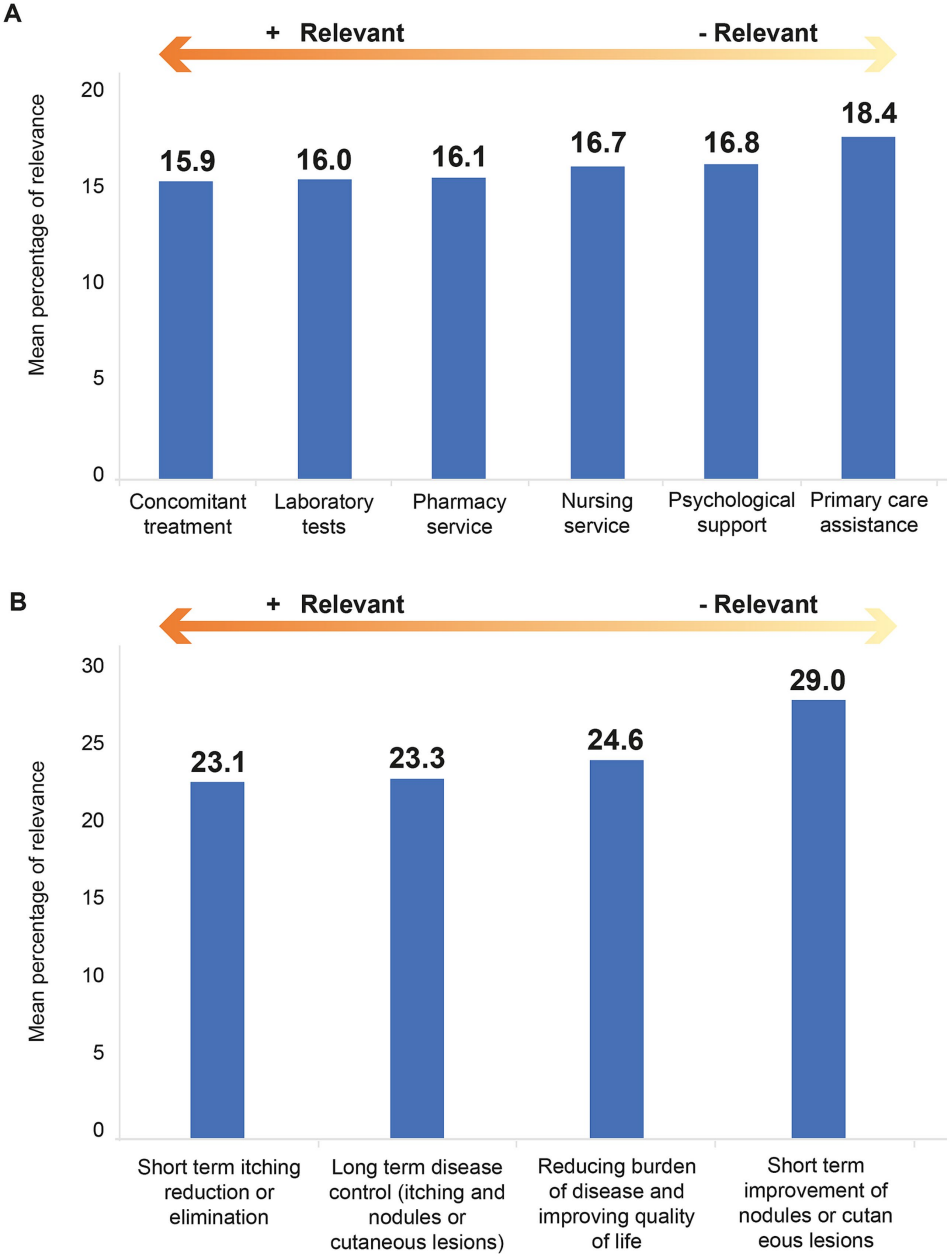
Relevance of the healthcare resources in the management of CPN **(A)** and relevance of the treatment goals for CPN management **(B)**, according to the participants (*N* = 39). The score assigned is higher the lower the preference for the valued item.

Physicians performed blood tests in 100% of their patients, biopsies in 69.2%, and chest X-rays in 23.1%. The mean time elapsed between the diagnosis of CPN and the start of treatment was 1.7 (SD 3.1) years (*N* = 37). Patients, on average, attended follow-up visits every 13.9 (SD 13.4) weeks, and a mean of 52.9% (SD 29) of cases required treatment modification (*N* = 38). Based on the participants’ experience, concomitant treatment was considered the most relevant healthcare resource in the management of CPN, while primary care was perceived as the least relevant (Figure 1A). The most relevant treatment goal for CPN was the short-term reduction or elimination of itching, while the least important was the short-term improvement of nodules or skin lesions (Figure 1B).

**Figure 2:**
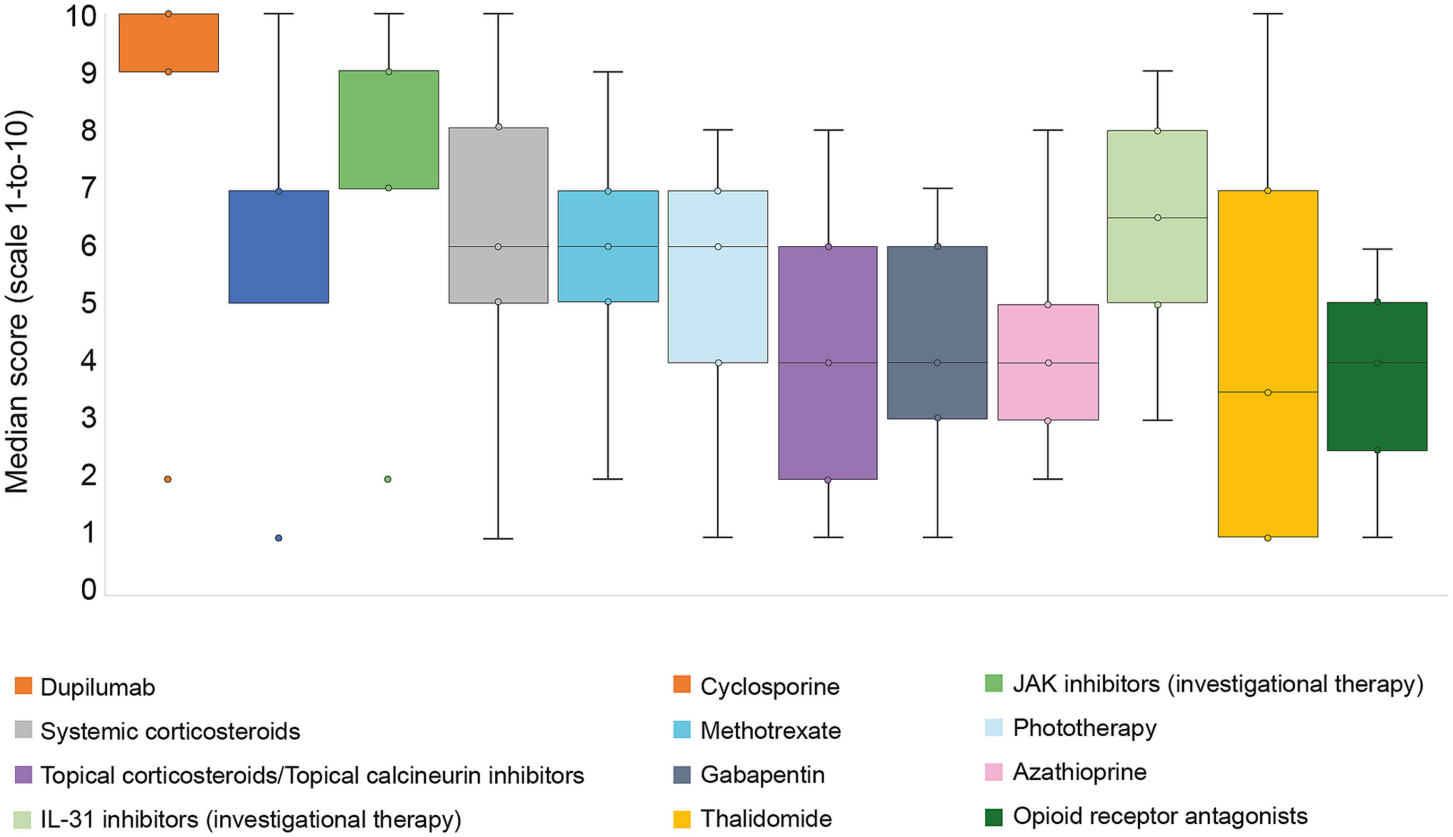
Effectiveness described for immunological and neuronal systemic therapies, according to the participants (*N* = 39), on a 1-to-10 scale. The boxplot represents the median, interquartile range Q1–Q3 (IQR), minimum, and maximum. Score 1 indicates the worst effectiveness, and 10 indicates the best effectiveness. IL, interleukin; JAK, Janus kinase.

Disease severity was considered the most important factor in determining treatment choice, according to the mean (SD) percentage of responses obtained by the participants (86.8% [SD 21.8]), followed by the type of symptoms (63.6% [SD 32.4]), atopic comorbidities (57.7% [SD 31.7%]), age (51.4% [SD 34.5]), non-atopic comorbidities (50.8% [SD 26.5]), concomitant psychiatric disorders (49% [SD 28.6]), and lesion type (45.1% [SD 34.5]). Dermatologists reported that a mean of 91.2% (SD 14.9) of patients were treated with topical corticosteroids and topical calcineurin inhibitors (Table 3). Among the patients with CPN receiving systemic immunological treatment, the most frequently used therapy was dupilumab, administered in a mean of 51.8% (SD 30.4) of cases (Table 3). Dupilumab was also considered the most effective treatment, with a median (IQR) score of 9 (9–10) on a 1–10 scale, where 10 represents the highest effectiveness (Figure 2). Other systemic treatments were used less frequently (Table 3).

**Table 3:** Treatments prescribed to patients with CPN.

Number of dermatologists	*N* = 39^1^ mean % (SD)
Topical corticosteroids/calcineurin inhibitors	91.2 (14.9)
Topical emollient	83.3 (25.7)
Systemic immunological treatment	48.4 (28.9)
Dupilumab	51.8 (30.4)
Cyclosporine	20.8 (22.9)
Methotrexate	12.9 (15.6)
Others	5 (11.6)
JAK inhibitors (investigational therapy)	4.5 (10.7)
Azathioprine	1.4 (4.1)
IL-31 inhibitors (investigational therapy)	1 (4.5)
Systemic neuromodulator treatment	15.7 (17)
Others	27.4 (41.3)
Gabapentin	26 (38.2)
Thalidomide	1.7 (7.1)
Opioid receptor antagonists	0.8 (2.7)
Kappa opioid receptor agonist	0 (0)
Systemic corticosteroids	22.9 (23.8)
Phototherapy	21 (22.5)

SD, standard deviation; JAK, Janus kinase; IL, interleukin; ^1^Number of dermatologists answering these questions.

## 4 Discussion

CPN is a complex and under-recognized disease, with a high symptom burden and limited therapeutic options. Until recently, its management has been based on clinical observations and published case series (19). In 2024, a clinical algorithm was developed to streamline the diagnostic process for patients with CPN (20), emphasizing a multidisciplinary approach to CPN patient management. The ECOSPIN study offers a comprehensive insight into the clinical landscape and management of CPN in Spain from the perspective of dermatologists.

The patient profile—as described by the participants—was predominantly female, aged between 51 and 60 years old, consistent with existing literature (21), and characterized by a longstanding disease (mean disease duration of >4 years). Pruritus was reported as the dominant symptom in the majority of cases, leading to the development of skin nodules (22). Indeed, in our setting, long-term disease control and management of nodules were important treatment goals, according to the participants. Notably, nodules were present in more than 82% of cases, but 18% lacked them, despite nodules being considered the hallmark of the disease. This discrepancy raises concerns about the huge heterogeneity in clinical presentation, as several types of lesions have been described in the European consensus (23). Additionally, it raises concerns about diagnostic heterogeneity, possible inclusion of early or atypical CPN phenotypes, or misclassification (2), and the insufficient application of proposed diagnostic criteria or overlap with other pruritic dermatoses.

The predominant lesion distribution in the lower legs and the upper back aligns with widely recognized patterns (1). The spectrum of lesions, including papules, excoriations, scars, and nodules, underscores the phenotypic heterogeneity and emphasizes the need to adopt subclassification strategies that go beyond nodular morphology alone (23). In addition, the majority of cases were perceived by dermatologists as having a dermatological origin, followed by psychological or psychosomatic causes. While the role of psychological stress is well defined in CPN pathogenesis, the distinction between causality and consequence is still unclear. It is known that stress plays a role in the development of several skin diseases by increasing sensory innervation, production of pruritogenic mediators, and lowering the itch threshold; however, the stigma associated with such conditions can also increase psychological burden (24). Indeed, more than half of the patients described by the dermatologists suffered from anxiety or depression. Paradoxically, dermatologists considered psychological support among the least relevant resources for CPN management. This miscorrelation reflects a critical need for increasing awareness of psychological burden and for psychodermatological collaboration (17). Furthermore, only 48% of dermatologists routinely assessed the quality of life using validated scales, highlighting a need for improved healthcare resources and reduced time pressures during consultations to facilitate patient-centered care.

Surprisingly, systemic and neurological comorbidities, such as hepatic, renal, or neuropathic conditions, were underrepresented in our study compared with previously published CPN cohorts (5, 25, 26). This may indicate insufficient multidisciplinary assessment, limited diagnostic workup in clinical practice, or underappreciation of systemic involvement. Kwatra et al. proposed two distinct CPN endotypes, the inflammatory and non-inflammatory, based on cytokine expression profiles, each associated with a different spectrum of comorbidities (27). More recently, Bao et al. described distinct comorbidity patterns in atopic and non-atopic CPN, with atopic patients exhibiting a higher prevalence of cardiovascular, neurological, and gastrointestinal conditions (28). Cardiometabolic comorbidities, such as arterial hypertension and dyslipidemia, were also common in our cohort, underscoring the systemic inflammatory nature of CPN. As reflected by the participants, the most common atopic comorbidity was AD (41%), and, although the mechanistic association between atopy and CPN is still unclear, there is increasing evidence that points toward shared pathophysiological pathways and potential bidirectional interactions (29, 30).

Regarding the management of patients with CPN, a mean delay of 1.7 years (SD 3.1) was observed between diagnosis and reported treatment initiation. This timeframe was based on dermatologists’ recall of when they commenced “symptomatic treatment for CPN management” following formal diagnosis. The term may have been interpreted variably across participants, potentially referring to specific CPN-directed therapies, such as topical corticosteroids, calcineurin inhibitors, systemic agents, or phototherapy, rather than general emollients. This delay may reflect diagnostic challenges, treatment access barriers, reimbursement restrictions, or initial management with supportive care before escalating to targeted therapies. The large standard deviation (3.1 years) suggests substantial heterogeneity in clinical practice patterns. This is consistent with previous reports from France showing a time to diagnosis exceeding 24 months (31). Contributing factors may include diagnostic uncertainties, reimbursement limitations, or a lack of awareness of the condition’s severity among some clinicians. Once treatment was initiated, among systemic agents, dupilumab was the most frequently prescribed (52%) and considered by dermatologists as the most effective treatment. Other systemic therapies, including cyclosporine, methotrexate, and phototherapy, were used to a lesser extent. Our data align with a 2024 network meta-analysis, which identified dupilumab as the preferred option for CPN given its efficacy in improving skin lesions, reducing pruritus, its favorable long-term safety profile, and its positive impact on patients’ quality of life (32). Interestingly, more than 25% of patients were treated with gabapentinoids, and a small proportion were treated with thalidomide. While these drugs are associated with safety concerns, their continued use and inclusion in the American and (IFSI) International Forum for the Study of Ich consensus treatment algorithms highlight the need to address the neuronal aspect of CPN pathophysiology (33). Safety and efficacy profiles in elderly patients with multiple comorbidities are critical, and treatments should have minimal pharmacokinetic interactions with other commonly prescribed medications, a particularly important consideration in this population (34). Furthermore, given the role of neuroinflammation in both pruritus generation and the development of skin lesions, therapies targeting only the inflammatory component may be insufficient for some patients (22).

In terms of therapeutic priorities, our study found that long-term disease control and skin lesions, along with short-term itch reduction, were important treatment goals for dermatologists. Certainly, insufficient focus on the structural and fibrotic components may contribute to lesion persistence and relapse (35). Not only is CPN a challenging condition in terms of patient management and treatment, but it also imposes a significant burden on the healthcare system. A notable proportion of the patients required unplanned visits, consultations with other healthcare providers, and psychological support, as reported by the participants. Consistent with previous studies, our data reveal a substantial use of healthcare services and a high economic burden, particularly among those requiring advanced therapies (25, 36–38).

This study has some limitations that should be considered. Its ecological design relies on aggregated, physician-reported data rather than individual patient-level data, which may affect the interpretation of our conclusions. Data collection was based on dermatologists’ recollections, which could be influenced by recall bias and subjective interpretation. The aggregated nature of the responses may limit detailed statistical analyses and the ability to establish direct associations between variables. A potential selection consideration is that dermatologists reported on their “last 5 patients with CPN,” which reflects the patient profile typically managed in specialized dermatology settings in Spain but may not represent the entire spectrum of CPN severity in the general population. This study was designed to characterize the clinical reality of dermatology consultations rather than provide population-level epidemiological data, which would require different methodological approaches.

This study also has some important strengths. A heterogeneous group of participants was recruited, ensuring a diverse sample across Spain with substantial clinical experience and a sufficient volume of patients. This approach allowed the collection of valuable real-world data on diagnostic protocols, treatment regimens, and management strategies and enabled the assessment of current challenges in daily clinical practice.

The results of this study confirm the prototypical profile of a middle-aged female with a substantial clinical and psychological burden, highlighting the high impact of CPN on the quality of life and the clear need for a multidisciplinary approach for a proper assessment and management. Considerable variability in diagnostic criteria, comorbidity identification, and therapeutic approaches was observed, reflecting the complexity of the disease. While dupilumab appears to be changing the treatment landscape of CPN, many unmet needs remain. Future research should focus on improving patient diagnostic approaches, enhancing multidisciplinary assessment, optimizing comorbidity identification, establishing accurate phenotypic identification and classification, and tailoring treatment to optimize patient outcomes.

## Data Availability

The raw data supporting the conclusions of this article will be made available by the authors without undue reservation.
