# Supplementary material for: Experience in the management of real-world patients with chronic prurigo nodularis in a dermatology setting: results from the ECOSPIN Spanish survey-based study
Source: Front Med (Lausanne). 2026 Apr 14;13:1769149. doi: 10.3389/fmed.2026.1769149 (PMC13120902; doi:10.3389/fmed.2026.1769149)
Supplement: Supplementary file 1 [file Supplementary_file_1.docx]

Supplementary Material

**Supplementary table. ECOSPIN Study Group.**

| Autonomous Community | Participant |
| --- | --- |
| Andalusia | Amalia Serrano Gotarredona |
|  | Isabel María Coronel Pérez |
|  | Javier Jesús Domínguez Cruz |
|  | José Juan Pereyra Rodríguez |
|  | Trinidad Montero Vílchez |
| Aragon | Ignacio Hernández Aragüés |
| Asturias | Jorge Santos-Juanes Jiménez |
| Balearic Islands | María Rosa Perelló Alzamora |
| Canary Islands | Alicia González Quesada |
|  | Francisco José Guimerá Martín-Neda |
| Cantabria | Marcos Antonio González López |
| Castile and Leon | Jesús Vega Gutiérrez |
| Catalonia | Antonio Guilabert Vidal |
|  | Esther Serra Baldrich |
|  | Ignasi Figueras Nart |
|  | María Constanza Riquelme Mc Loughlin |
|  | Mónica Munera Campos |
|  | Vicente Expósito Serrano |
| Galicia | Ángeles Flórez Menéndez |
|  | Rosa María Fernández Torres |
| La Rioja | Íñigo Martínez de Espronceda Ezquerro |
| Madrid (Community of) | Álvaro Iglesias Puzas |
|  | Araceli Sánchez Gilo |
|  | Cristina Mauleón Fernandez |
|  | Francisco Javier Ortiz de Frutos |
|  | María Bibiana Pérez García |
|  | Natalia Hernández Cano |
|  | Pablo Chicharro Manso |
|  | Susana Córdoba Guijarro |
| Murcia (Region of) | Francisco José De León Marrero |
| Basque Country | Irene García Río |
|  | Maider Pretel Irazábal |
|  | María Salomé Álvarez Sánchez |
| Valencian Community | Francisco Javier Miquel Miquel |
|  | José María Sánchez Motilla |
|  | Juan Francisco Silvestre Salvador |
|  | Mercedes Rodríguez Serna |
|  | Sergio Santos Alarcón |
|  | Violeta Zaragoza Ninet |

**Supplementary data. Study questionnaire.**

INSTRUCTIONS

The study questionnaire is organized in five different sections:

1. Healthcare professional data: in this section you must include your sociodemographic data and the information related to your professional profile and experience.
2. Sociodemographic and clinical profile of patients and characterization of disease: in this section you must answer to questions about sociodemographic and clinical characteristics of CPN patients, including comorbidities, and CPN-related characteristics, including family history, disease duration. signs and symptoms at diagnosis, aetiology of CPN, lesion location, and disease severity.
3. Impact of disease burden on CPN patients: in this section you must answer to questions related to the emotional burden, sleep disruption, interference with daily life activities, and quality of life of CPN patients.
4. Management of CPN patients: in this section you must answer to questions about the management of CPN in terms of diagnosis, treatment, and follow-up in routine clinical practice at Dermatology consultations.
5. Impact of disease burden on healthcare resources: in this section you must answer to questions related to the use of direct and indirect healthcare resources in CPN patients in routine clinical practice.

The questionnaire contains different fields which must be filled as follows:

- Open fields (“___ “) must be completed with numbers or words
- Single-response fields (“o”) must be clicked to select a single option.
- Multiple-response fields (“□”) must be clicked to select one or more of the provided options.

IMPORTANT: please remember that all questions must be filled. For those questions that you consider, please enter “0” where applicable. *It is highly recommended to save the questionnaire through its fulfilment.*

QUESTIONNAIRE

**PARTICIPANT ELIGIBILITY CRITERIA**

At least 5 years of experience (as post-MIR dermatologist “board-certified):

- Yes
- No

To see a mean of 3 patients with CPN (including patients newly diagnosed and under follow-up) per month in the last year:

- Yes
- No

**HEALTHCARE PROFESSIONAL DATA**

1. Please, enter your age:

____ years old

1. Please, select your sex:

- Male
- Female

1. Please select the type of hospital/center where you develop your professional activity:

- General Hospital
- Specialized Hospital
- University/Academic/Tertiary Hospital

1. Please select the type of hospital/center where you develop your professional activity regarding its public or private nature:

- Public Hospital
- Private Hospital
- Public and Private Hospital (*both models)*

1. Please select the autonomous community to which your work canter belongs:

- Andalucía
- Aragón
- Asturias
- Baleares
- Canarias
- Cantabria
- Castilla-La Mancha
- Castilla y León
- Cataluña
- Ceuta (Ciudad Autónoma)
- Extremadura
- Galicia
- La Rioja
- Madrid (Comunidad)
- Melilla (Ciudad Autónoma)
- Murcia
- Navarra
- País Vasco
- Valenciana (Comunidad)

1. Please, select the years of experience in your therapeutic area (dermatology):

- From 5 to 10 years
- From 11 to 19 years
- Equal or over 20 years

1. Please, indicate the average number of patients attending your office per week:

____

1. Please, indicate the current percentage of patients diagnosed with CPN in relation to the total number of patients with dermatological diseases attending your office (at the time of the survey):

____ %

1. Please, indicate the total number of patients who have been diagnosed with CPN (new cases) attending your office in the last 12 months:

____

**SOCIODEMOGRAPHIC AND CLINICAL PROFILE OF CPN PATIENTS AND CHARACTERIZATION OF DISEASE**

To complete the information required in this section, please consider the **aggregate data** of the **last 5 patients with CPN seen in your office**. Please do not evaluate individual patients, but provide a general trend that, based on your experience and protocol with these patients as a group, may be representative of your usual clinical practice. You should try to ensure that the data you provide is as close as possible to what has occurred in your usual clinical practice.

1. Regarding the last 5 CPN patients attending your office, please, specify their age:

- <20 years: _____ %
- 21-30 years: _____%
- 31-40 years: ______%
- 41-50 years: _____ %
- 51-60 years: _____ %
- 61-70 years: _____ %
- 71-80 years: ______ %
- >81 years: _____ %

1. Regarding the last 5 CPN patients attending your office, please, specify their sex (if none, please enter 0%):

- Male: _____ %
- Female: _____ %

1. Regarding the last 5 CPN patients attending your office, please, specify the percentage of patients presenting with the following concomitant atopic comorbidities (if none, please enter 0%):

- Atopic dermatitis: _____ %
- Asthma: _____ %
- Chronic rhinosinusitis with nasal polyps (CRSwNP):_____ %
- Chronic spontaneous urticaria (CSU):_____ %
- Eosinophilic esophagitis: ____ %
- Chronic obstructive pulmonary disease (COPD): _____ %
- Bullous Pemphigoid (BP): ____ %
- Allergic rhinitis (nasal mucosa inflammation): ___ %
- Allergic conjunctivitis (eye inflammation): ___%

Please note that patients may have more than one comorbidity, therefore, the percentages provided may sum up more than 100%

1. Regarding the last 5 CPN patients attending your office, please, specify the percentage of patients presenting with the following comorbidities (if none, please enter 0%):

- Arterial hypertension: _____ %
- Dyslipidemia: _____ %
- Obesity: _____ %
- Metabolic syndrome: _____ %
- Cardiovascular disease: ____ %
- Gastrointestinal disorders: ____ %
- Renal disease: ____ %

Please note that patients may have more than one comorbidity, therefore, the percentages provided may sum up more than 100%

1. Regarding the last 5 CPN patients attending your office, please, specify the percentage of patients with family history of CPN (if none, please enter 0%):

_____ %

- Unknown data

1. Regarding the last 5 CPN patients, please, specify the average time of disease duration since diagnosis (if none, please, enter 0):

_____ months

*_____* years

- Unknown data

1. Regarding the last 5 CPN patients, please, specify the percentage of patients presenting with the following clinical signs at diagnosis of CPN (if none, please enter 0%):

- Nodules: _____ %
- Papules: _____ %
- Plaque lesions _____ %
- Ulcerative lesions: _____ %
- Excoriations: _____ %
- Scars: _____ %
- Lichenification: _____ %
- Dyspigmentation: _____ %
- Other: _____ %

Please note that patients may have more than one sign, therefore, the percentages provided may sum up more than 100%

1. Regarding the last 5 CPN patients, please, specify the percentage of patients presenting with the following symptoms at diagnosis of CPN (if none, please enter 0%):

- Itching: _____ %
- Burning: _____ %
- Pain: _____ %
- Sleep loss: _____ %

Please note that patients may have more than one symptom, therefore, the percentages provided may sum up more than 100%

1. Regarding the last 5 CPN patients attending your office, please, specify the percentage of patients presenting with the following emotional symptoms at diagnosis of CPN related to the disease (if none, please enter 0%):

- Depression: _____ %
- Anxiety: _____ %
- Other emotional symptoms: ___%

Please note that patients may have more than one symptom, therefore, the percentages provided may sum up more than 100%

1. Regarding the last 5 CPN patients attending your office, please, specify the aetiology of CPN (if none, please enter 0%):

- Dermatological: _____ %
- Systemic (i.e., DM, infections, liver / renal insufficieny): _____ %
- Neurological (i.e., herpes zoster, restless legs syndrome): _____ %
- Psychological / psychosomatic (i.e., depression, anxiety, obsesive-compulsive behaviour, tactile hallucinations): _____ %
- Tumoral (skin cancer, hematological malignancies, solid tumors): ____ %
- Medication: _____ %
- Unknown Aetiology: _____ %

Please note that patients may have more than one comorbidity, therefore, the percentages provided may sum up more than 100%

1. Regarding the last 5 CPN patients, please, specify CPN lesion location (if none, please enter 0%):

- Face: ____ %
- Scalp: ____ %
- Anterior lower legs: _____ %
- Posterior lower legs: _____ %
- Anterior forearms: _____ %
- Posterior forearms: _____ %
- Hands: _____ %
- Upper back: _____ %
- Abdomen: _____ %

Please note that patients may have more than one lesion location, therefore, the percentages provided may sum up more than 100%

1. Regarding the last 5 CPN patients, please, specify CPN severity based on the average worse itch numeric rating scale (NRS) score during the last week (if none, please enter 0%):
   - Range 0-3: ______%
   - Range 4-6: ______%
   - Range 7-10: _____%
2. Regarding the last 5 CPN patients attending your office, please, specify the investigator´s global scale (IGA) score:

Activity:

- 0: _____ %
- 1: _____ %
- 2: _____ %
- 3: _____ %
- 4: _____ %

Nodules: _____ %

o NA/Unknown

1. According to your experience and clinical practice, please, rate (from 1 to 10, with 1 being the less important and 10 being the most important) the relevance of the following factors to determine prognosis and evolution of CPN:

- Age: ____
- Type of symptoms at diagnosis: ____
- Lesion type at diagnosis: ____
- Disease severity at diagnosis: ____
- Concomitant disease: ____
- Concomitant psychiatric disorders: ____
- Genetic factors: ____

**IMPACT OF DISEASE BURDEN ON CPN PATIENTS**

To complete the information required in this section, please consider the **aggregate data** of the **last 5 patients with CPN seen in your office**.

1. Regarding the last 5 CPN patients attending your office, please, specify the percentage of patients who needed psychologic/behavioural support (i.e., related to anxiety or depression) (if none, please, enter 0%):

_____ %

- Unknown data

1. Regarding the last 5 CPN patients, specify the percentage of patients presenting with sleep disruption due to CPN (if none, please, enter 0%):

_____ %

- Unknown data

1. Regarding the last 5 CPN patients, please, specify the percentage of patients reporting poor quality of life due to CPN-related issues (if none, please, enter 0%):

_____ %

- Unknown data

1. Regarding the last 5 CPN patients, specify the percentage of patients in whom quality of life is assessed using questionnaires/scales in your routine clinical practice (i.e. DLQI) (if none, please, enter 0%):

_____ %

- Unknown data

**MANAGEMENT OF CPN PATIENTS**

To complete the information required in this section, please consider the **aggregate data** of the **last 5 patients with CPN seen in your office**.

1. Regarding the last 5 CPN patients, please, specify the average time elapsed from diagnosis of CPN to the start of symptomatic treatment for CPN management (if none, please enter 0):

_____ days

_____ months

- Unknown data

1. Please, prioritize the goals of treatment for CPN management from 1 as the most relevant goal to 4 as the less relevant goal:

- Short term itching reduction or elimination: ___
- Short term improvement of nodules or cutaneous lesions: ____
- Long term disease control (itching and nodules or cutaneous lesions): ____
- Reducing burden of disease and improving quality of life: ____

Please note the number assigned to each option should be different (1, 2, 3, or 4)

1. Regarding the last 5 CPN patients, please, specify the percentage of patients receiving the following therapies (if none, please enter 0%)

- Topical emollient: _____%
- Topical corticosteroids/Topical calcineurin inhibitors: ____%
- Systemic corticosteroids: _____%
- Immunological systemic: _____ %
- Neuronal systemic _____%
- Phototherapy: ____%

Please note that patients may receive more than one type of treatment,

1. Please, specify the factors that, in your experience and clinical practice determine the selection of therapy of CPN (if none, please enter 0%):

- Age: _____ %
- Atopic comorbidities: _____ %
- Non atopic comorbidities: _____ %
- Concomitant psychiatric disorders: _____ %
- Type of symptoms: _____ %
- Lesion type: _____ %
- Disease severity: _____ %

Please note that more than one factor can be selected, therefore, the percentages provided may sum up more than 100%

1. Regarding the CPN patients receiving immunological systemic treatment, please, specify the percentage of patients receiving the following therapies (if none, please enter 0%):

- Cyclosporine: ____%
- Methotrexate: ____%
- Azathioprine: ____ %
- Dupilumab: ____%
- IL-31 inhibitors (Investigational therapy): ___%
- JAK inhibitors (Investigational therapy): ___%
- Other: ____ %

Please note that patients may receive more than one type of treatment

1. Regarding the CPN patients receiving neuronal systemic treatment, please, specify the number of patients receiving the following therapies (if none, please enter 0%):

- Gabapentin: ____ %
- Thalidomide: ____ %
- Opioid receptor antagonists: ____ %
- Kappa opioid receptor agonist: ____ %
- Other: ____ %

1. According to your experience and clinical practice, rate (from 1 to 10, with 1 being the worse effectiveness and 10 being the best effectiveness) the effectiveness that you consider associated to the following therapies (if no clinical experience, please enter 0):

- Topical corticosteroids/Topical calcineurin inhibitors: _____
- Systemic corticosteroids: _____
- Cyclosporine: ____
- Methotrexate: ____
- Azathioprine: ____
- Dupilumab: ____
- IL-31 inhibitors (Investigational therapy): ____
- JAK inhibitors (Investigational therapy): ____
- Gabapentin: ___
- Thalidomide: ____
- Opioid receptor antagonists: ___
- Phototherapy: ____

1. Regarding the last 5 CPN patients, please, specify the mean frequency of the follow-up visits for disease evaluation:

- Every __ weeks
- Unknown data

1. Regarding the last 5 CPN patients, please, specify the procedures/test performed for CPN follow-up assessment, including lab monitoring for the treatment (if none, please enter 0):

- Blood test (i.e., platelet count, liver function: AST, ALT): _____
- Biopsy: ____
- Chest X-Ray: ____

Please note that more than one test can be selected

1. Regarding the last 5 CPN patients, please, specify the percentage of patients who required treatment modification (if none, please, enter 0%):

______ %

- Unknown data

**IMPACT OF DISEASE BURDEN ON DIRECT HEALTHCARE RESOURCES**

To complete the information required in this section, please consider the **aggregate data** of the **last 5 patients with CPN seen in your office**.

1. Regarding the last 5 CPN patients attending your office, please, specify the percentage of patients who required additional non-planned visits (without previous appointment; either due to symptoms related to CPN or complications) due to CPN evolution during the last year (if none, please enter 0):

_____ %

- Unknown data

1. Regarding the last 5 CPN patients, please, specify the percentage of patients who required visits to other healthcare providers during the last year (if none, please enter 0):

_____

- Unknown data

1. Regarding the last 5 CPN patients, please, specify the percentage of patients who required hospitalization due to CPN during the last year (if none, please enter 0):

_____

- Unknown data

1. Regarding the last 5 CPN patients, please, specify the percentage of patients who needed psychologist support / behavioural or emotional support (i.e., related to anxiety or depression) during the *last year* (if none, please, enter 0):

_____

- Unknown data

1. Please, specify the healthcare resources you consider more relevant for CPN management in routine clinical practice by scoring in order of importance, from 1 being the most relevant to 6 being the less relevant:

- Laboratory tests: ____
- Concomitant treatment: ____
- Nursing service: _____
- Pharmacy service: _____
- Primary care assistance: _____
- Psychological support: _____

Please note the number assigned to each option should be different (1, 2, 3, 4, 5 or 6)
